# Characterization of the Chromosome 4 Genes That Affect Fluconazole-Induced Disomy Formation in *Cryptococcus neoformans*

**DOI:** 10.1371/journal.pone.0033022

**Published:** 2012-03-07

**Authors:** Popchai Ngamskulrungroj, Yun Chang, Bryan Hansen, Cliff Bugge, Elizabeth Fischer, Kyung J. Kwon-Chung

**Affiliations:** 1 Molecular Microbiology Section, Laboratory of Clinical Infectious Diseases, National Institute of Allergy and Infectious Diseases, National Institutes of Health, Bethesda, Maryland, United States of America; 2 Department of Microbiology, Faculty of Medicine, Siriraj Hospital, Mahidol University, Bangkok, Thailand; 3 Electron Microscopy Unit, Rocky Mountain Laboratories, National Institute of Allergy and Infectious Diseases, National Institutes of Health, Hamilton, Montana, United States of America; 4 FEI Company, Hillsboro, Oregon, United States of America; Yonsei University, Republic of Korea

## Abstract

Heteroresistance in *Cryptococcus neoformans* is an intrinsic adaptive resistance to azoles and the heteroresistant phenotype is associated with disomic chromosomes. Two chromosome 1 (Chr1) genes, *ERG11*, the fluconazole target, and *AFR1*, a drug transporter, were reported as major factors in the emergence of Chr1 disomy. In the present study, we show Chr4 to be the second most frequently formed disomy at high concentrations of fluconazole (FLC) and characterize the importance of resident genes contributing to disomy formation. We deleted nine Chr4 genes presumed to have functions in ergosterol biosynthesis, membrane composition/integrity or drug transportation that could influence Chr4 disomy under FLC stress. Of these nine, disruption of three genes homologous to Sey1 (a GTPase), Glo3 and Gcs2 (the ADP-ribosylation factor GTPase activating proteins) significantly reduced the frequency of Chr4 disomy in heteroresistant clones. Furthermore, FLC resistant clones derived from *sey1Δglo3Δ* did not show disomy of either Chr4 or Chr1 but instead had increased the copy number of the genes proximal to *ERG11* locus on Chr1. Since the three genes are critical for the integrity of endoplasmic reticulum (ER) in *Saccharomyces cerevisiae*, we used Sec61ß-GFP fusion as a marker to study the ER in the mutants. The cytoplasmic ER was found to be elongated in *sey1Δ* but without any discernable alteration in *gcs2Δ* and *glo3Δ* under fluorescence microscopy. The aberrant ER morphology of all three mutant strains, however, was discernable by transmission electron microscopy. A 3D reconstruction using Focused Ion Beam Scanning Electron Microscopy (FIB-SEM) revealed considerably reduced reticulation in the ER of *glo3Δ* and *gcs2Δ* strains. In *sey1Δ*, ER reticulation was barely detectable and cisternae were expanded extensively compared to the wild type strains. These data suggest that the genes required for maintenance of ER integrity are important for the formation of disomic chromosomes in *C. neoformans* under azole stress.

## Introduction

Cryptococcosis is caused by two environmental basidiomycetous yeasts, *Cryptococcus neoformans* and *Cryptococcus gattii* [1]. The infection caused by both species is most commonly treated with amphotericin B as an induction regimen followed by azoles for long-term maintenance therapy [2]. Azole drugs target the biosynthetic pathway of ergosterol that is an essential component of the fungal membrane [2], [3]. The ergosterol biosynthetic pathway has been extensively characterized (for review see [2]. It is synthesized in the endoplasmic reticulum (ER) starting from acetyl-CoA through a series of enzymes encoded by different *ERG* genes [2]. The sterol is then delivered to the cell membrane via both vesicular and non-vesicular routes [3], [4].

Since its introduction in 1990, fluconazole (FLC) has been the most widely used azole antifungal as a prophylactic regimen as well as for maintenance therapy of yeast infections such as candidiasis and cryptococcosis [5], [6], [7]. Long-term treatment with FLC results in the emergence of drug resistance strains. The mechanism of FLC resistance has been well characterized in the pathogenic yeast, *Candida albicans* (for review see [8], [9], [10]. Mutations or over-expression of enzymes in the ergosterol synthetic pathway [11], [12], [13], [14] or efflux pumps [15], [16], [17], [18] are known to be major causes in the emergence of azole resistant strains. Since the number of drug resistant cases reported in cryptococcosis patients has been much less than in candidiasis patients, the mechanism of drug resistance in *C. neoformans* has not been widely investigated. Like in other fungi, a mutation in *ERG11* was reported from an azole resistant clinical strain and over expression of the ABC transporter, *AFR1* was reported to cause resistance to FLC in a strain of *C. neoformans* constructed in the laboratory [19], [20].

In 1999, azole resistance due to an unknown mechanism termed ‘heteroresistance’ was reported in both serotype A and D strains of *C. neoformans* isolated from patients in Italy and Israel [21]. Recently, the heteroresistance was reported to be an intrinsic mechanism of adaptive resistance to triazoles in every strain of *C. neoformans* and *C. gattii* [21], [22], [23]. The phenomenon of heteroresistance was characterized as the emergence of minor subpopulations within a single colony of the susceptible strain that could adapt to FLC concentrations higher than the strain's minimum inhibitory concentration (MIC). The acquired resistance in these subpopulation is lost upon release from drug stress [23]. The lowest concentration of FLC at which resistant subpopulation emerges was defined as the level of heteroresistance to fluconazole (LHF) of each strain. The FLC resistant subpopulations that emerged at the LHF (32 µg/ml) for the genome sequenced strain H99 were found to mostly contain disomic Chr1 [24]. As the FLC concentration was elevated, however, additional disomies were observed for Chr4, at 64 µg/ml and Chr10, Chr14 [24] and Chr3 at 128 µg/ml [unpublished data]. The extra copies of Chr1 and Chr4 formed in response to azole stress were lost upon daily transfer in drug free media [24]. Chr1 contains two genes known to be involved in azole resistance in *C. neoformans*, *ERG11*, the target of fluconazole, and *AFR1*, a drug transporter [24]. Elevation of the dosage of these genes as a result of the disomy appears to have enabled the strains to overcome the drug stress.

Apart from these two genes, factors mandating disomy formations of the other chromosomes have yet to be identified. Here, we show that Chr4 is another chromosome most frequently found to be disomic at drug concentrations higher than the LHF. We focused on the identification of Chr4 genes which contribute to the strains' drug resistance and necessitate formation of disomy at higher concentration of FLC. Chr4 of *C. neoformans* does not contain genes that are involved in ergosterol biosynthesis but does contain two ABC transporters and a homolog of *PDR16*. Deletion of the two ABC transporters had no effect on azole resistance while deletion of *PDR16* reduced the level of azole resistance. Interestingly, *PDR16* did not play a significant role in Chr4 disomy formation. However, genes on Chr4 which are required for maintenance of the endoplasmic reticulum (ER) integrity were found to affect disomy formation under FLC stress.

## Results

### High frequency of Chr4 disomy at elevated concentrations of FLC

**Figure 1:**
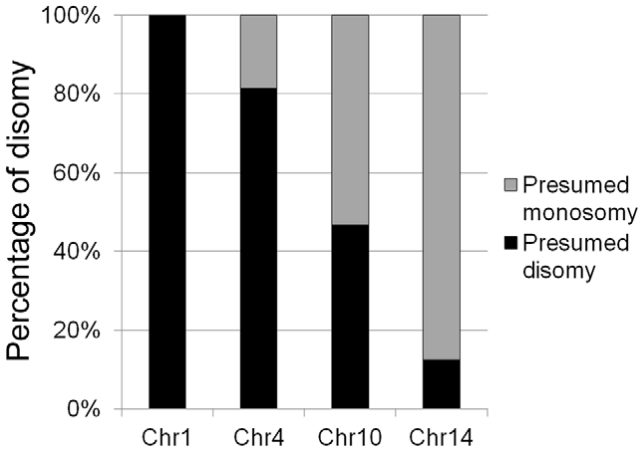
Chr4 disomy is associated with survival of *C. neoformans* at high concentrations of FLC. 16 resistant clones grown at 128 µg/ml FLC (3LHF) were randomly selected and the numbers of each chromosome in the genome were estimated by measuring the copy number of the genes specific to each chromosome. The histogram displays the percentage of presumed disomy for each chromosome in the 16 clones.

The heteroresistance phenotype was identified as a fraction of the cell population that could grow on media containing a concentration of FLC that is higher than the strain's MIC [23]. For convenience, in this report we have defined the first level of heteroresistance to FLC (1LHF) as the lowest concentration of FLC at which minor resistant subpopulations emerge. We used arbitrary 2-fold increments of FLC concentration to define the subsequent LHF's. For instance, the 1LHF of the wild type strain H99 is 32 µg/ml at which Chr1 is disomic in a majority of the clones [24]. In the previous study, disomies of Chr1 and Chr4 were most commonly found in H99 at the second LHF (2LHF, 64 µg/ml) while those other chromosomes such as Chr10 and 14 were observed disomic at 128 µg/ml FLC (3LHF) [24]. We have examined several independent FLC resistant clones of the H99 strain derived from 2LHF and 3LHF to determine the frequency at which these chromosomes were duplicated in response to FLC stress. By using the quantitative real time PCR (qPCR) to determine copy-number of the genes, we found 100% of the 9 FLC resistant clones isolated from 2LHF contained disomic Chr1 while 55% of these clones also contained disomic Chr4 (data not shown). At 3LHF, Chr1 was consistently duplicated in every clone while Chr4 was the second most frequently duplicated chromosome in the 16 independent FLC resistant clones (82%) (Figure 1). This suggests an importance of the Chr4 disomy for the survival of *C. neoformans* at very high concentrations of FLC.

### Identification of the genes potentially attributable to formation of Chr4 disomy under fluconazole stress

One likely reason for the occurrence of Chr4 disomy in strains resistant to high FLC concentrations is that some genes located on Chr4 play an important role in azole resistance and an increase in their copy number enhances tolerance for elevated azole stress. To address this hypothesis, we screened annotated genes on Chr4 and selected total nine genes that are likely to be non-essential and are associated with drug resistance. The nine genes selected and deleted were in two categories: 1) Genes reported to be important for antifungal resistance included: the putative ABC transporters (*CNAG_07799* and *CNAG_05150*) [25], homolog of *PDR16* (pleiotropic drug resistance, *CNAG_04984*) [26]; 2) Since FLC is known to perturb the cell membrane, the second category included those genes functionally associated with ergosterol synthesis or membrane composition/integrity included: *LRO1* (diacylglycerol acyltransferase, *CNAG_05152*) [27], *TLK1* (phosphatidylinositol 3 kinase, *CNAG_05220*) [28], [29], *SEY1* (GTPase with a role in ER morphology, *CNAG_05124*) [30], *SLC1* (1-acyl-sn-glycerol-3-phosphate acyltransferase, *CNAG_05247*) [31], and *GCS2* and *GLO3* (ADP-ribosylation factor GTPase activating proteins, *CNAG_05050* and *CNAG_05314*) [32], respectively. No known homologs of *ERG* genes are present on Chr4.

**Figure 2:**
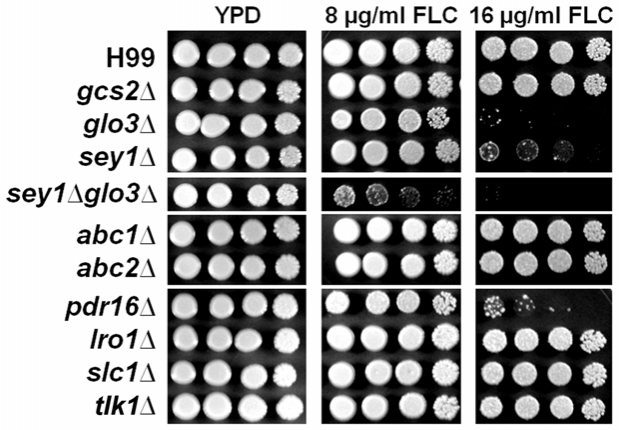
Spot assay for FLC tolerance. Cells of the indicated strains were spotted on YPD alone or YPD supplemented with either 8 µg/ml or 16 µg/ml FLC and incubated at 30°C for 5 days. Increase in sensitivity to FLC was seen in strains containing disruptions of several genes on Chr4 including, *GLO3*, *SEY1* and *PDR16*.

We first examined the importance of these genes in FLC tolerance by spot assays on FLC containing media. We determined homologs of *SEY1* and *GLO3* to be important for FLC tolerance since deletion of *sey1* and *glo3* resulted in the decrease of 1LHF (the lowest level of FLC at which heteroresistant clones emerge) from 32 µg/ml to 16 µg/ml (Figure 2). As observed in *Candida albicans* [33], disruption of *PDR16* compromised the ability of cells to tolerate FLC at 16 µg/ml (Figure 2). Unlike *AFR1*, the ABC transporter on Chr1 that exerts a major impact on FLC tolerance [24], the two ABC transporters on Chr4 did not affect FLC tolerance (Figure 2). Furthermore, deletion of the other five genes also did not affect FLC tolerance in H99. Double deletions of *SEY1* and *GLO3*, functionally redundant with *GCS2*, resulted in a more severe susceptibility to FLC in which 1LHF was decreased to 8 µg/ml compared to 32 µg/ml in H99. (Figure 2 and Table 1). Since several attempts to generate a double deletant of *gcs2Δglo3Δ* failed, It is possible that deletion of these two genes which encoding proteins associated with ER trafficking is lethal in *C. neoformans* as is the case in *S. cerevisiae* [32]. The wild type level of FLC tolerance was restored in all susceptible mutants complemented with the wild type genes (Figure S1).

**Table 1:** List of strains and their levels of heteroresistance.

Strain	Description	Concentration of FLC at 1, 2 and 3LHF (µg/ml)
H99	wild type	32, 64, 128
*gcs2Δ*	ADP-ribosylation factor GTPase activating protein	32, 64, 128
*glo3Δ*	ADP-ribosylation factor GTPase activating protein	16, 32, 64
*sey1Δ*	GTPase interacts with ER-shaping protein	16, 32, 64
*sey1Δglo3Δ*	double deletion of *SEY1* and *GLO3*	8, 16, 32
*abc1Δ*	ATP binding cassette transporter	32, 64, 128
*abc2Δ*	ATP binding cassette transporter	32, 64, 128
*pdr16Δ*	pleiotropic drug resistance	16, 32, 64
*lro1Δ*	diacylglycerol acyltransferase	32, 64, 128
*slc1Δ*	1-acyl-sn-glycerol-3-phosphate acyltransferase	32, 64, 128
*tlk1Δ*	PIK-related protein kinase	32, 64, 128
*gcs2Δ::GCS2* *	homologous complementation with *GCS2*	32, 64, 128
*glo3Δ::GLO3*	homologous complementation with *GLO3*	32, 64, 128
*sey1Δ::SEY1*	homologous complementation with *SEY1*	32, 64, 128
*sey1Δglo3Δ::SEY1*	homologous complementation with *SEY1*	16, 32, 64
*sey1Δglo3Δ::GLO3*	homologous complementation with *GLO3*	16, 32, 64
*sey1Δ,SEY1chr3*	complementation to Chr3 with *SEY1*	32, 64, 128
*glo3Δ,GLO3chr3*	complementation to Chr3 with *GLO3*	32, 64, 128

* The *GCS1* homolog of *S. cerevisiae* was named *GCS2* as *GCS1* has already been used to designate glucosylceramide synthase in *C. neoformans* [62].

### Deletion of genes encoding the proteins required for maintenance of ER morphology and trafficking affects the frequency of Chr4 disomy formation

**Figure 3:**
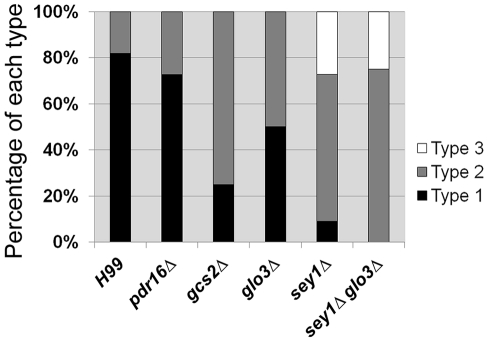
Frequency of Chr4 disomy formation in deletants of Chr4 genes. Clones resistant to 3LHF derived from each deletant were isolated and the copy number of Chr1 and Chr4 in the genome was inferred by assessing the copy number of the genes specific to each chromosome by qPCR. The figure displays the percentage of each type of disomy in the FLC resistant strains. Type1 denotes strains that contain Chr1 and Chr4 disomy, Type2 denotes strains that contain Chr1 disomy, Type3 denotes strains that contain no disomic Chr1 or Chr4.

Since *glo3Δ*, *sey1Δ* and *pdr16Δ* showed a reduction in FLC tolerance, we studied the effect of each gene deletion in the disomy formation of Chr4. Furthermore, since Chr4 disomy was more frequently detected at 3LHF in H99 (Figure 1), we analyzed clones from each of the deletant that emerged at 3LHF. Quantitative PCR was carried out using probes specific for genes on each chromosome to evaluate the status of presumed Chr4 disomy. Interestingly, only one among 11 FLC resistant clones (9%) derived from *sey1Δ* and 50% in *glo3Δ* (6/12) expressed a presumed disomy of Chr4 while it was 82% (9/11) in the wild type strain and 73% (8/11) in the *pdr16Δ* strain (Figure 3). Furthermore, deletion of the ABC transporters had no affect the Chr4 disomy formation (data not shown). Unexpectedly, in 3 of the 11 FLC resistant clones (27%) derived from *sey1Δ* did not show disomies of either Chr1 or Chr4 (Type 3 in Figure 3, Type 1 denotes strains that contain Chr1 and Chr4 disomy, Type 2 denotes strains that contain Chr1 disomy only, Type 3 denotes strains that do not contain disomies of either Chr1 or Chr4). The clones that did not contain disomies of either Chr1 or Chr4 (Type 3) showed similar comparative genome hybridization (CGH) patterns for all chromosomes as the wild type. The Type 3 clones were most likely the result of mutation(s) at other loci relevant for drug resistance since they remained resistant to FLC despite continuous passages in drug free media for 3 months (data not shown).

Although deletion of *GCS2*, a gene on Chr4 that shares a function similar to that of *GLO3* in other organisms [32], [34], did not affect FLC tolerance but only 2 of 8 *gcs2Δ* derived FLC resistant clones (25%) showed disomic Chr4 (Figure 3). Interestingly, all FLC resistant clones derived from the double deletants of *sey1Δglo3Δ* lacked Chr4 disomy (Figure 3). Since the classification of three types based on disomic status of Chr1 and Chr4 was presumed by qPCR, CGH was also performed for at least one clone from each type in every deletant to determine whether gene duplication represented duplication of the entire chromosome (Figure S2). The CGH results were consistent with qPCR data except for the Type 1 clones derived from the double deletants (see below). Furthermore, the original frequency of Chr4 disomy was restored in *sey1Δ*, *glo3Δ*, and *gcs2Δ* mutants when reconstituted with the respective wild type genes (Figure S3). These results suggest that the genes that encode proteins required for putative ER structure and trafficking affect Chr4 disomy formation in *C. neoformans*.

### Relocation of *SEY1* and *GLO3* to Chr3 increases the frequency of Chr3 disomy

**Figure 4:**
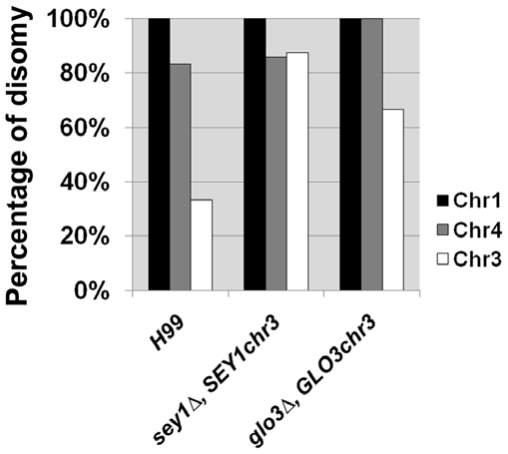
Frequency of Chr3 disomy is increased in the strains with *SEY1* and *GLO3* translocation to Chr3. The wild type genes *SEY1* and *GLO3* were each inserted into Chr3 of the respective deletant strains *sey1Δ* and *glo3Δ*. 6–8 resistance clones grown at 3LHF were analyzed for copy number of the genes specific to each chromosome. Each bar represents the frequency of disomy formation in each chromosome.

A correlation between the chromosomal location of genes specifically important for azole resistance and disomy of the corresponding chromosome has been shown in *C. albicans* and *C. neoformans* [24], [35]. For example, relocation of the *ERG11* gene from Chr1 to Chr3 in *C. neoformans* resulted in disomy of Chr3 instead of Chr1 at the 1LHF [24]. To determine a similar importance of the *SEY1* and *GLO3* genes on chr4, we reconstituted the *sey1Δ* and *glo3Δ* strains independently by inserting a wild type copy of the respective genes into Chr3 instead of their native location on Chr4. When clones resistant at 3LHF were analyzed, the frequency of Chr3 disomy was significantly higher in both reconstituted strains *SEY1* and *GLO3* compared to the wild type strain (Figure 4). Interestingly, however, the frequency of Chr4 disomy in these strains was comparable to the wild-type strain.

### Only a segment of Chr1 is duplicated in *sey1Δ* derived double deletants

**Figure 5:**
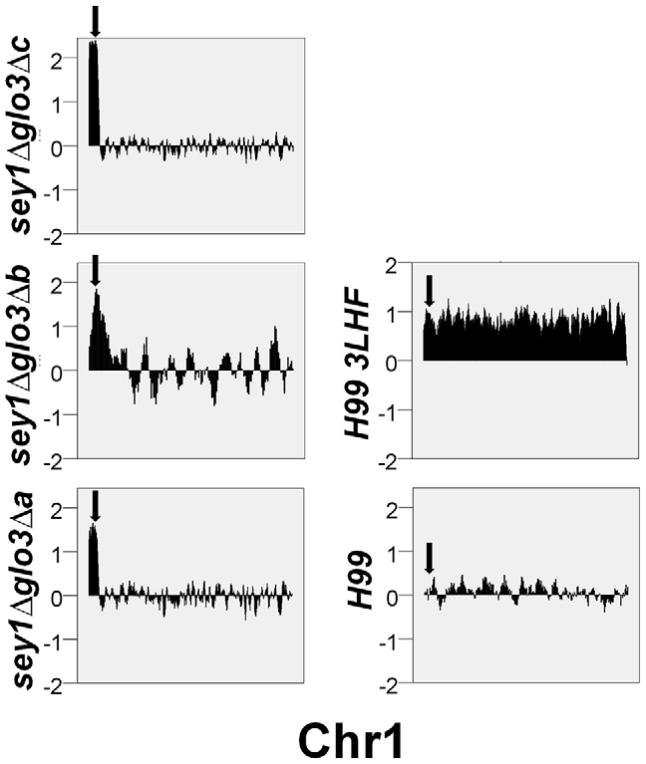
CGH analysis of the *sey1Δglo3Δ* double deletants isolated from 3LHF. CGH was performed with genomic DNA extracted from double deletants that showed Type 2 duplication patterns at 3LHF in Figure 3. H99 and the resistant clones of H99 grown at 3LHF were included as controls. Only the Chr1 status is shown. No additional disomy was found in any of the clones except for the clone from H99 at 3LHF (data not shown). Arrows indicate the location of *ERG11*. Each bar represents the copy number of each gene residing on Chr1 or 4 in log_2_ scale.

**Figure 6:**
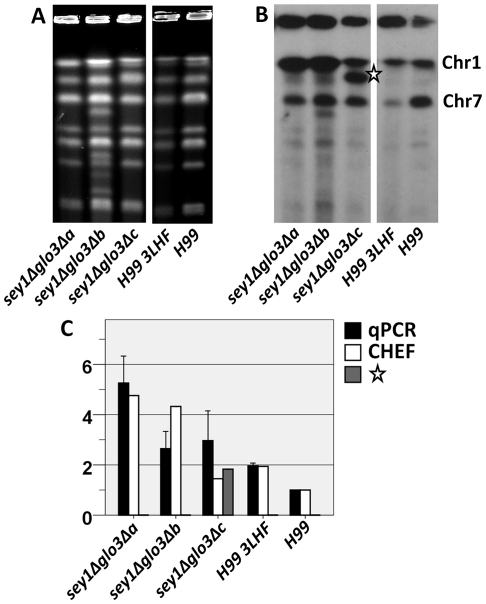
Analysis of the *ERG11* chromosomal location in the Type2 *sey1Δglo3Δ* clones. **A**) CHEF gel analysis. Chromosomes of the indicated strains were separated by CHEF and stained with ethidium bromide. **B**) Southern blot analysis. DNA of the CHEF gel was transferred to membrane and hybridized with *ERG11* (Chr1) and *CNAG_06646* (Chr7) probes. Star indicates the non-Chr1 located *ERG11*. **C**) Copy-number of *ERG11*. White bar indicates the copy number of *ERG11* from the CHEF quantified by phosphor-imager and the black bar represents the copy number of *ERG11* analyzed by qPCR. Gray bar = non-Chr1 *ERG11* indicates the copy number of *ERG11* from the CHEF quantified by phosphor-imager of the band marked with a star from B). Error bar = 2 SD.

Based on the qPCR of genes present on Chr1 and Chr4 , Type2 FLC resistant clones derived from *sey1Δglo3Δ* only contained disomy of chr1 (Figure 3). However, the CGH patterns observed in these clones were unusual in that instead of the whole chromosome being duplicated, there seem to have increased gene dosage only at the chr1 region proximal to the *ERG11* locus (Figure 5). Interestingly, the region where gene duplication occurred in Chr1 is distant from the putative centromere region and no gene duplication was observed in any other chromosome (data not shown). Furthermore, some of the clones had more than two copies of *ERG11*. It is possible that the extra copies of the Chr1 genes could have resulted from partial chromosome duplication, gene duplication combined with chromosomal translocation, or from an episomal event. To investigate the chromosomal status of these clones, we examined their genome by clamped homogeneous electric field (CHEF) gel analysis. One of the clones (*sey1Δglo3Δb*) clearly contained additional chromosomes compared to the wild type (Figure 6A). However, using *ERG11* as a probe for the CHEF blot, the hybridization signal indicated that *ERG11* is localized only on Chr1 in *sey1Δglo3Δb* and not on the extra chromosomes. Two other double deletant clones did not show any change in the chromosomal pattern. The *ERG11* signal was only detected in Chr1 of *sey1Δglo3Δa*. Additionally, the *ERG11* gene in the *sey1Δglo3Δc* clone was present on Chr1 as well as on another chromosome (Figure 6B). Copy number of the *ERG11* gene in each clone determined by CHEF blot quantitated by phosphoimager agreed with the qPCR results (Figure 6C). This result indicates that the increase in copy number demonstrated by qPCR is not contributed by an episomal event. Therefore, the genomic regions that contain the extra copies of *ERG11* revealed in CGH were likely to have resulted from a partial duplication of Chr1 and/or a chromosomal translocation.

### Deletants of the genes controlling ER morphogenesis show variable degrees of aberration in ER morphology

**Figure 7:**
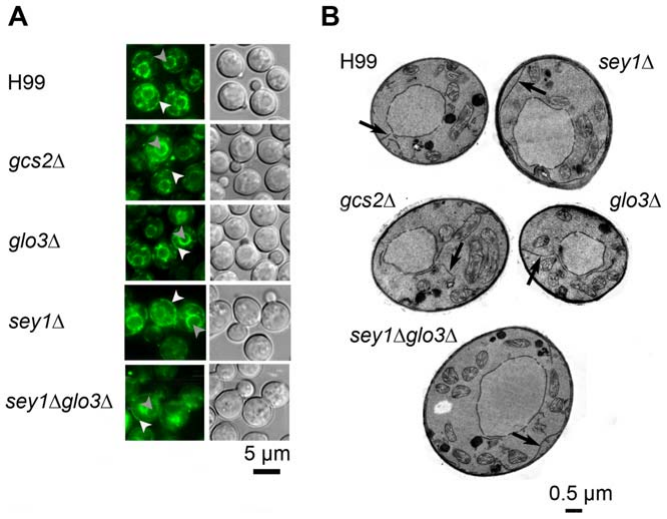
Disruption of *SEY1, GCS2 and GLO3* causes ER elongation. **A**) A GFP fusion of the ER protein Sec61βp was expressed in each indicated strain. Cells from fresh overnight culture grown on YPD agar at 30°C were visualized under a florescent microscope. White arrow head = ER; grey arrow head = nucleus (determined by co-localized Hoechst 3342 dye staining; Figure S4). **B**) Transmission Electron Microscopy (TEM) of each deletant and the wild type cells. Cells were grown overnight on a shaker at 30°C in YPD broth to mid log phase and cells were prepared for and examined by TEM.

Since *SEY1, GLO3 and GCS2* are known to be important for ER morphogenesis in other organisms [30], [32], [36], [37], we thought it was possible that deletants of these genes in *C. neoformans* may have structural changes in ER. We constructed a GFP fusion with the *C. neoformans* ER located protein, Sec61ß and used the construct to monitor the ER morphology. Transformants of Sec61ß-GFP in the wild type strain showed the GFP signal to be located near the nuclear envelope and the element near the plasma membrane connected with strands in the cytoplasm; a typical ER pattern as seen in *S. cerevisiae* (Figure 7A) [38]. By contrast, the cytoplasmic ER in the *sey1Δ* strain was elongated which was not the case in the *gcs2Δ* and the *glo3Δ* strains. Elongation of the ER was also evident in the double deletants of *sey1Δglo3Δ* (Figure 7A). The morphological differences of the ER between the wild type and the deletant strains were better illustrated in the TEM where the cytoplasmic ER was elongated in all the mutants compared to the wild type strain (Figure 7B, arrows).

### 3D reconstruction using Focused Ion Beam-Scanning Electron Microscope showed double deletants lost the reticular structure of normal ER

**Figure 8:**
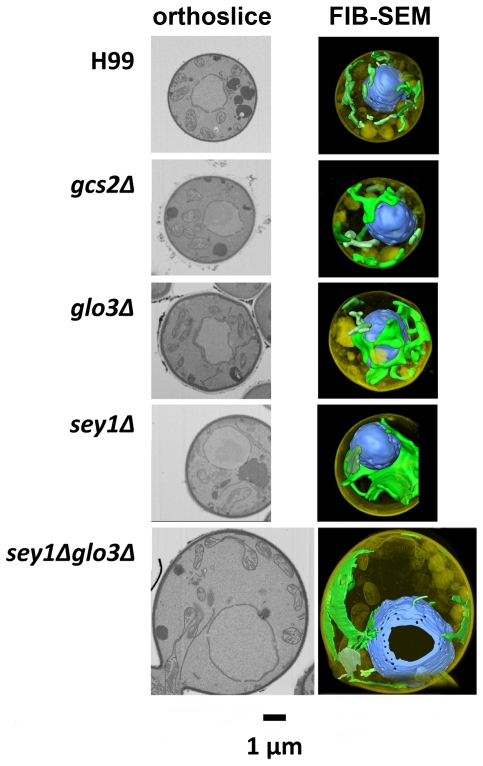
The FIB-SEM showed all mutants to have variable degrees of aberration in ER morphology. Left column shows orthoslices representing central slices through the volume. 3D reconstructions of the ER in indicated strains were generated from data collected by serial 3–10 nm cuts of each sample through the cell. ER and nuclei were psuedo-colored with green and blue color, respectively. The FIB-SEM picture of H99 was taken at different angle from the orthoslice to have a clearer view of the ER structure.

Since ER in eukaryotic cells is a reticular structure [30], [39] which extends from the nuclear membrane into the cytoplasm, a typical TEM section of the cells does not lend itself to visualization of the whole ER structure in a three dimensional context. Thus, we used the FIB-SEM technology to generate serial section views for 3D reconstruction of cryptococcal cells. The wild type cells showed the typical cisternae (continuous sheet) with reticular structures extending throughout the cytoplasm in 3D (Figure 8). Compared to the wild type, *gcs2Δ* and *glo3Δ* showed reduced reticular ER structures. Most strikingly, in *sey1Δ* and *sey1Δglo3Δ* cells, the reticular ER morphology was barely detectable and formed mostly expanded cisternae distinct from the wild type pattern (Figure 8). These data indicated that *C. neoformans* genes *SEY1*, *GLO3*, and *GCS2* are important for maintaining normal ER morphology.

## Discussion

The relationship between occurrence of aneuploidy and azole resistance has been well documented in both pathogenic yeasts, *C. albicans* [40] and *C. neoformans* [24]. Formation of either partial or whole chromosome disomy has been shown to benefit the yeasts for survival under azole stress [24], [41]. In addition to the importance of *ERG11* and *AFR1* for disomy formation in Chr1 for survival under azole stress in *C. neoformans*, we present here an association between three genes that encode proteins involved in ER trafficking and structural maintenance with formation of disomy when stressed with high levels of FLC. Our results show that disruptions of *SEY1*, *GCS2*, and *GLO3* result in significantly reduced frequencies of Chr4 disomy. Moreover, translocating some of these genes to Chr3 increased the frequency of disomy formation in Chr3 at high FLC levels.

Since formation of disomy is an energy consuming process and its maintenance would be stressful for the cells, aneuploidy is typically transient. Survival in a stressful environment such as that presented by high drug concentrations, only cells that possess either duplicated whole chromosomes or part of the chromosome containing genes pertinent to drug resistance [42]. For example, *ERG11* which encodes the azole target protein is localized on Chr5 in *C. albicans* [35], [40] and on Chr1 in *C. neoformans* [24] and these chromosomes are found either duplicated partially or entirely in the FLC resistant clones which emerged in response to azole stress. We demonstrate here that a new set of genes related to ER function play an important role in Chr4 disomy formation in *C. neoformans*. Of the 9 Chr4 genes studied, the deletion of 2 of these genes, *sey1Δ* and *glo3Δ*, resulted in higher susceptibility to FLC and lower frequency of disomies. Although deletion of the *GCS2* gene did not alter the strain's sensitivity to FLC, it did affect Chr4 disomy formation at high concentrations of FLC. Interestingly, *PDR16* was required for FLC resistance but did not have a significant effect on the frequency of Chr4 disomy formation. This suggests that Chr4 disomy formation does not solely depend on the genes that affect FLC susceptibility. Moreover, the location of genes on certain chromosomes influences the outcome of disomy formation since translocation of *SEY1* or *GLO3* to Chr3 results in an increase in the frequency of Chr3 disomy formation. However, in spite of such relocation of one or the other gene, the frequency of Chr4 disomy in these strains was maintained to wild type levels. This suggests that *SEY1* and *GLO3* are equally important for disomy of Chr4 and disomy of Chr4 is not dependent on the location of *SEY1* or *GLO3* but rather the presence of functional copies in the genome. Duplications of other Chr4 genes together with either *SEY1* or *GLO3* appear to be beneficial for the survival of *C. neoformans* under FLC stress.

Moreover, the influence of *SEY1* and *GLO3* on formation of Chr4 disomy can be explained. The absence of either of the two genes renders *C. neoformans* unable to resist FLC stress. Hence, increase in the copy number of Chr4 genes resulting from disomy would not enable *C. neoformans* to reach a resistance level required to overcome the heightened drug stress. This explanation is supported by the induction of Chr3 disomy as *SEY1* and *GLO3* are relocated to Chr3. These results suggest that the elevated expression of these genes resulting from increased copy number is important for *C. neoformans* to tolerate azole stress. However, the mechanism is probably more complicated since the deletion of *GCS2* genes also compromised Chr4 disomy formation without significant perturbation of ER integrity or azole susceptibility and warrants a further study.

The FIB-SEM study shows that the ER morphology is altered in all three mutant strains. In particular, reticulation of the ER in the double mutant *sey1Δglo3Δ* was greatly reduced and the cisternae expanded significantly. These data suggest that *SEY1*, *GLO3* and *GCS2* are important for the ER integrity. The involvement of *SEY1* and *GLO3* in ER morphology has been demonstrated in other systems. In *S. cerevisiae*, Sey1 is a GTPase and is involved in the generation of the tubular ER network [30]. Glo3 is an ADP-ribosylation factor GTPase activating proteins involved in ER-Golgi transport which shares functional similarity with Gcs1p. Double deletion of *GLO3* and *GCS1* (homolog of *GCS2* in *C. neoformans*) causes morphological aberrations under restricted conditions [32], [37]. Furthermore, in *S. cerevisiae*, *GCS1* and *GLO3* mediates Golgi-ER and post-Golgi vesicle [32], [43], [44]. Thus, we tested functions of the two secreted cryptococcal virulence factors, urease [45] and laccase [46]. No differences in secretion of the two virulence factors were found among the mutants comparing to H99 (Figure S5). This suggests that defects on vesicle transport of those mutants are minimal. However, it is known that intact protein transport between ER and Golgi is required for protein glycosylation [47] and disruption of *GLO3* or *GCS1* impairs glycosylation in *S. cerevisiae* [32]. This is also the case in *C. neoformans* where disruption of either *GLO3* or *GCS1* causes increases in sensitivity to tunicamycin (a N-glycosylation inhibitor [48], [49]) (Figure S6). It has been known that intact glycosylation is required for both mitosis [50] and cytokinesis [51] but N-glycosylation has not been reported to be associated with mitosis. How N-glycosylation is associated with disomy formation remains unexplained and need further study.

Although disomy formation was observed in Chr4 and other chromosomes, Chr1 was the only chromosome consistently found to be duplicated at high levels of FLC in all our studies. This could be due to the selection for survival under FLC stress where *ERG11/AFR1* duplication enables the strains to effectively tolerate high FLC concentrations. Interestingly, we observed that the FLC resistant clones derived from *sey1Δglo3Δ* double deletions failed to produce duplication of the entire chromosome, but only caused a partial duplication of the chromosome region proximal to *ERG11* locus. These results underscore the importance of the *ERG11* gene and the necessity of ER structural integrity for the survival of *C. neoformans* under FLC stress.

Despite discoveries of disomy/isochromosome formation as a novel mechanism of azole resistance in pathogenic fungi [24], [40], the mechanism by which azoles cause such a phenomena remains unknown. Here, our results shed some light on the relationship between impairment of ER integrity and chromosome disomy formation under FLC treatment. Since FLC targets synthesis of ergosterol essential for fungal cells and the sterol is synthesized in ER before being delivered to the plasma membrane via both vesicular and non-vesicular routes [3], [4], it is conceivable that alterations in ER integrity would affect the survival of cells under FLC stress. But, how does ER influence disomy formation? The ER in eukaryotic cells is a dynamic and continuous membrane network consisting of interconnected cisternae and tubules in direct contact with the nuclear envelope, which stretches throughout the cytosol [39], [52]. In fact, the ER may influence mitosis through its interconnection with the nuclear envelope [52], [53], [54] which in turn controls chromosomal segregation through its association with the spindle pole body [55]. Furthermore, it has been shown that alterations of ER conformation could rescue the defect of nuclear envelope breakage in *pim1Δ* and allowed the cells to survive through mitosis [53]. Hence, disruption of ER function, such as glycosylation, combined with drastic change in ER structure may interfere with the normal pattern of chromosomal segregation and subsequently thwart chromosomal disomy formation. However, the mechanisms of how ER function/structure influence such events remains unclear and further studies are warranted to resolve this important question of cell biology.

## Materials and Methods

### Strains and Media

All deletants were constructed in the *C. neoformans* H99 background [56] and are listed in Table 1. All strains were maintained on non-selective YPD agar (1% yeast extract, 2% peptone, 2% glucose, 2% agar). YPD supplemented with 8, 16, 32, 64, or 128 µg/ml FLC or 125 ng/ml tunicamycin were used to study phenotypes of the deletants. Melanin and urease induction media was prepare according to a standard protocol [57]. For spot assays, 2 µl of a cell suspension with an optical density at 600 nm (OD_600_) of 2 to 3 or more 10-fold dilutions were spotted on YPD agar with or without the drugs. The plates were incubated at 30°C for 3–5 days and photographed.

### Gene manipulations

Annotated genes on Chr4 were selected from the H99 genome database (http://www.broadinstitute.org/annotation/genome/cryptococcus_neoformans/MultiHome.html). Genes of interest were disrupted by biolistic transformation [58]. Briefly, disruption constructs were created by overlapping PCR technique [59] or vector cloning linked with Nourseothricin (*NAT1*), G418 (*NEO*) or Hygromycin (*HGR*) resistance genes as dominant selectable markers. Then, the constructs were transformed into the H99 cells by a Bio-Rad model PDS-1000/He biolistic particle delivery system. Homologous integrations were confirmed by PCR and Southern hybridization. Complementation of each deletant was accomplished by homologous integration at the deleted locus or reintroduced to a specific location on Chr3 as described previously [24]. Primers for overlapping PCR and vector cloning are listed in Table S1.

### Quantitation of gene dosage

Quantitative real time PCR (qPCR) assays were performed to quantitate the gene copy number on specific chromosomes in wild-type, deletants and FLC-resistant strains as previously described [24]. 6–16 independent clones of each strain were tested.

### Comparative Genome Hybridization (CGH)

Genomic DNA was extracted using overnight culture in YPD as described previously [24]. DNA labeling was performed by BioPrime®Array CGH Genomic Labeling System Kit (Invitrogen, Carlsbad, CA) according to the manufacturer's instruction. Briefly, genomic DNA was digested by *Dpn*II (New England Biolabs, Ipswich, USA). Alexa647 and Alexa555 dye were labeled to the experimental and control samples, respectively. Subsequently, the labeled samples were hybridized to JEC21-based 70mer slides (http://genome.wustl.edu/services/microarray/cryptococcus_neoformans) and analyzed as previously described [24].

### Clamped homogeneous electric field (CHEF) gel electrophoresis

Cells were grown on YPD agar with or without FLC for 2–3 days. Cells were harvested from the plate and dilute to 2.0 OD_600_ in 10 ml of 10% β-mercaptoethanol and incubate at 37°C for 30 min. Cells were pelleted and resuspended in 1 ml of spheroplasting solution (1 M sorbitol, 10 mM EDTA, 100 mM sodium citrate, pH5.8) mixing with 300 mg/ml Vinoflow FCE (Novozymes, Bagsvaerd, Denmark) and incubated at 30°C for 1.5 hr with gentle shaking. After pelleting the cells by spinning at 700 g for 10 min and washing twice with cold spheroplasting solution, cells were resuspended in 150 µl of the spheroplasting solution. Gel preparation and electrophoresis condition were performed according to a previous study [60]. Subsequently, Southern hybridization was performed using ^32^P labeled probes of *ERG11* and the promoter region of *CNAG_ 06646* on Chr7 which is proven not to form disomy by CGH of any strain in this study (data not shown). After hybridization, the membranes were exposed to a phospho-imager screen and signals were quantified with ImageQuant (Molecular Dynamics). The relative copy number of each gene was obtained by comparing the signal intensity of the experimental probe, *ERG11*, to that of the internal control probe, *CNAG_ 06646*.

### Fluorescence microscopy

To visualize ER in the yeast, an ER-protein Sec61β/Sbh1 homolog, *CNAG_06351*, was identified through BlastP implemented in the H99 genome website and tagged with GFP [30]. A construct of Sec61β-GFP was cloned and transformed into indicated strains by biolistic transformation. The GFP signals were visualized for ER morphology under the fluorescence Zeiss Axiovert microscope and Axiovision (version 4.0) software.

### Transmission electron microscopy (TEM)

Cells were prepared and stained according to a previous publication [61]. Briefly, cells were grown in YPD broth overnight, diluted and transferred to fresh YPD and grown to mid-log phase. Cells were harvested and washed once in 1 ml of primary fixative (0.1 M sorbitol, 1 mM MgCl_2_, 1 mM CaCl_2_, 2% glutaraldehyde in 0.1 M PIPES buffer, pH 6.8). Then, cells were resuspended in the fixative. Cells were post-fixed with 2% KMnO_4_, dehydrated with a graded ethanol series and propylene oxide prior to infiltrating and embedding in Epon. Cells were prepared for visualization of membrane structures under transmission electron microscope (TEM) according to the method described [61]. Briefly, the fixed samples were sectioned using a Leica UC6 (Leica Microsystems, Vienna, Austria) and viewed on a Hitachi H-7500 (Hitachi, Tokyo, Japan) at 80 kV. Digital images were obtained with a Hammamatsu XR-100 digital camera system (AMT, Danvers, MA.)

### Focused Ion Beam-Scanning Electron Microscopy (FIB-SEM)

Using the same sample preparation technique noted above, specimens were mounted into the FIB-SEM. All data sets were collected on a FEI Helios NanoLab 650 DualBeam™ equipped with Ga^+^ LMIS FIB used for milling (FEI, Hillsboro, OR). Prior to milling a protective platinum cap was deposited on the area of interest and 3–10 nm cuts were made. To perform milling operation, the FIB was set to 30 keV and a beam current of 790 pA was employed. The pixel sizes and horizontal field width for each data set are as follows: 12 µm; *sey1Δglo3Δ* 3 nm×3 nm×5 nm, 13 µm; 20.4 µm; H99, *sey1Δ*, *glo3Δ*, and *gcs2Δ* 10 nm×10 nm×10 nm, 20.4 µm. All imaging was done at 2 keV and 200 pA. A BSE signal was collected with brightness and contrast inverted. Data was aligned using the Amira Visualization Package (version 5.3.3, Visage Imaging, Carlsbad, CA). Data was binned by 4 with the IMOD software package (version 4.2.10, University of Colorado Boulder, Boulder, CO) and all 3-D surface models were created from unfiltered images by manually selecting areas of interest and smoothing the 3-D volumes using Amira.

## Supporting Information

Figure S1 **Spot assay.** Cell suspensions of each indicated strain were spotted on different media and incubated at 30°C for 5 days. Reconstitution of the deletants by the wild type gene restored their susceptibility to FLC. sg = *sey1Δglo3Δ*.
(TIF)

Figure S2 **CGH analysis of disomy formation in each type.** Type1 = disomy of Chr1 and Chr4, Type2 = disomy of Chr1 only, Type3 = no disomy of either Chr1 or Chr4. Each panel is representative of the CGH result from each disomy type. Only the status of Chr1 and Chr4 copy number is shown. The CGH data is consistent with the qPCR results except for the double mutant. Each bar represents the copy number of each gene residing on Chr1 or 4 in log_2_ scale.
(TIF)

Figure S3 **The frequency of Chr4 disomy formation is restored in each mutant reconstituted with the wild type gene.** Clones of each complemented strain resistant at 3LHF were isolated and their Chr1 and Chr4 copy numbers were inferred by analyzing dosage of the genes specific to each chromosome by using qPCR. The figure displays the frequency of each f disomy type among the FLC resistant strains. Type1 denotes strains that contain disomies of Chr1 and Chr4, Type2 denotes strains that contain disomy of Chr1 only, and Type3 denotes strains containing no disomies of either Chr1 or Chr4.
(TIF)

Figure S4 **ER structure of H99.** A GFP fusion of the ER protein Sec61βp was expressed in the wild type. Cells from fresh overnight culture grown on YPD agar at 30°C were visualized under a florescent microscope. Nucleus is determined by co-localized Hoechst 3342 dye staining giving blue color. (From left to right; GFP, Hoechst 3342 staining, merge, DIC).
(TIF)

Figure S5 **Ability to produce urease and melanin was unchanged in all mutants.** Cell suspensions of each indicated strain were spotted on melanin (A) and urease (B) test media and incubated at 30°C for 2 days.
(TIF)

Figure S6 **Disruption of** ***GCS2*** **and** ***GLO3*** **increased sensitivity to tunicamycin, an ER perturbing agent.** Cells suspensions were serially diluted 10-fold from a stock at OD_600_ = 2 and spotted on YPD agar plates with or without 125 ng/ml tunicamycin. Plates were incubated at 30°C for 3 days.
(TIF)

Table S1 **Primers used.**
(DOCX)
