# Supplementary figures and images for: Characterization of the Chromosome 4 Genes That Affect Fluconazole-Induced Disomy Formation in Cryptococcus neoformans
Source: PLoS One. 2012 Mar 7;7(3):e33022. doi: 10.1371/journal.pone.0033022 (PMC3296764; doi:10.1371/journal.pone.0033022)

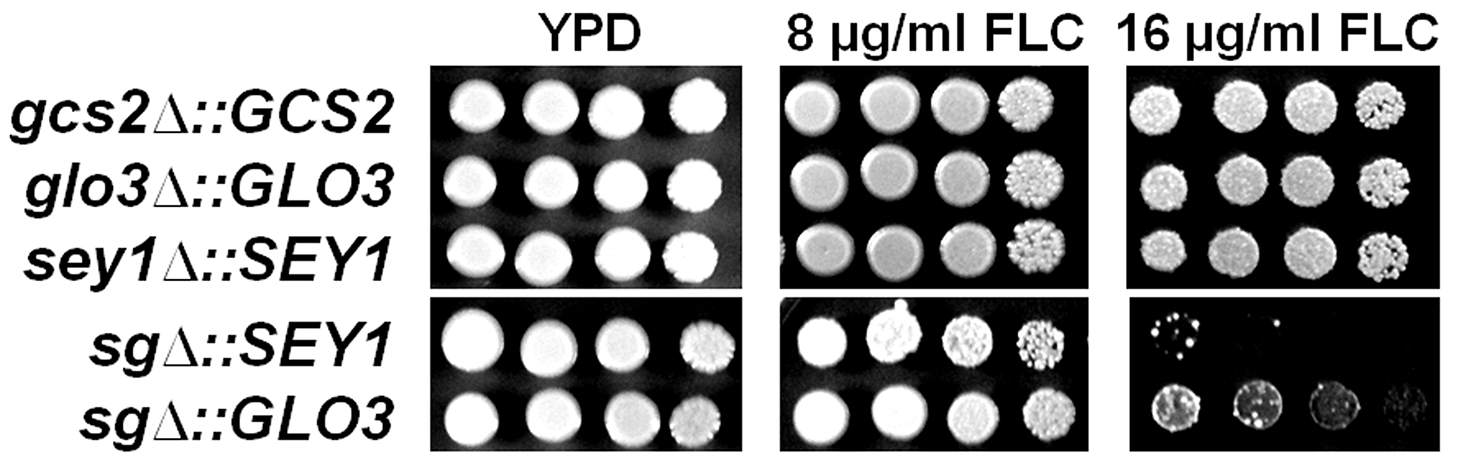

Supplement: Figure S1 — Spot assay. Cell suspensions of each indicated strain were spotted on different media and incubated at 30°C for 5 days. Reconstitution of the deletants by the wild type gene restored their susceptibility to FLC. sg = sey1Δglo3Δ. (TIF) [file pone.0033022.s001.tif]

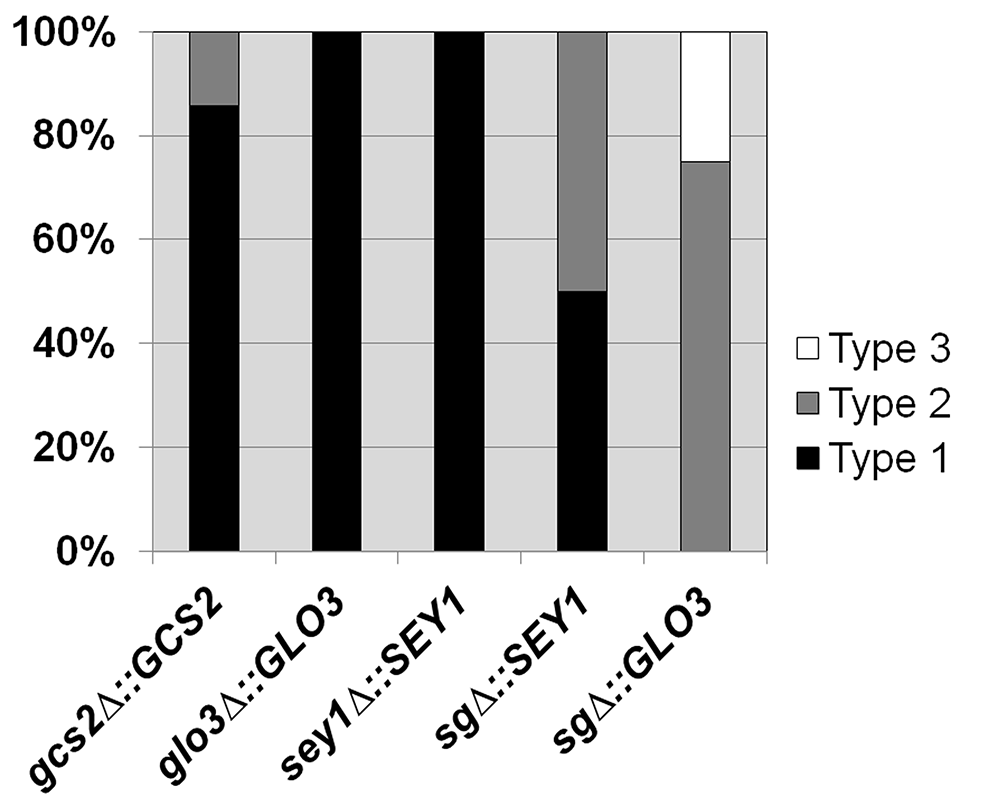

Supplement: Figure S2 — CGH analysis of disomy formation in each type. Type1 = disomy of Chr1 and Chr4, Type2 = disomy of Chr1 only, Type3 = no disomy of either Chr1 or Chr4. Each panel is representative of the CGH result from each disomy type. Only the status of Chr1 and Chr4 copy number is shown. The CGH data is consistent with the qPCR results except for the double mutant. Each bar represents the copy number of each gene residing on Chr1 or 4 in log2 scale. (TIF) [file pone.0033022.s002.tif]

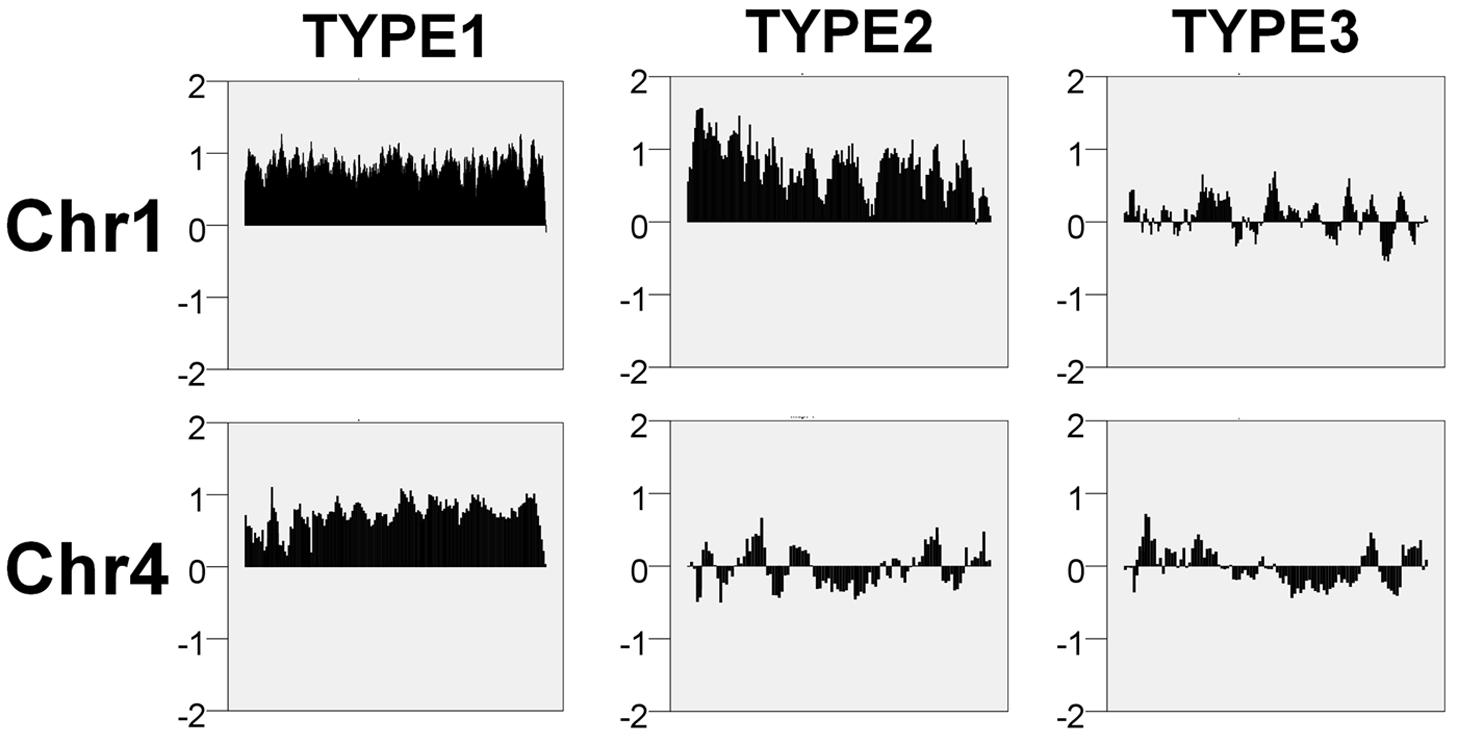

Supplement: Figure S3 — The frequency of Chr4 disomy formation is restored in each mutant reconstituted with the wild type gene. Clones of each complemented strain resistant at 3LHF were isolated and their Chr1 and Chr4 copy numbers were inferred by analyzing dosage of the genes specific to each chromosome by using qPCR. The figure displays the frequency of each f disomy type among the FLC resistant strains. Type1 denotes strains that contain disomies of Chr1 and Chr4, Type2 denotes strains that contain disomy of Chr1 only, and Type3 denotes strains containing no disomies of either Chr1 or Chr4. (TIF) [file pone.0033022.s003.tif]

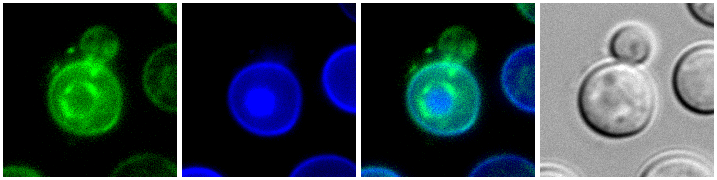

Supplement: Figure S4 — ER structure of H99. A GFP fusion of the ER protein Sec61βp was expressed in the wild type. Cells from fresh overnight culture grown on YPD agar at 30°C were visualized under a florescent microscope. Nucleus is determined by co-localized Hoechst 3342 dye staining giving blue color. (From left to right; GFP, Hoechst 3342 staining, merge, DIC). (TIF) [file pone.0033022.s004.tif]

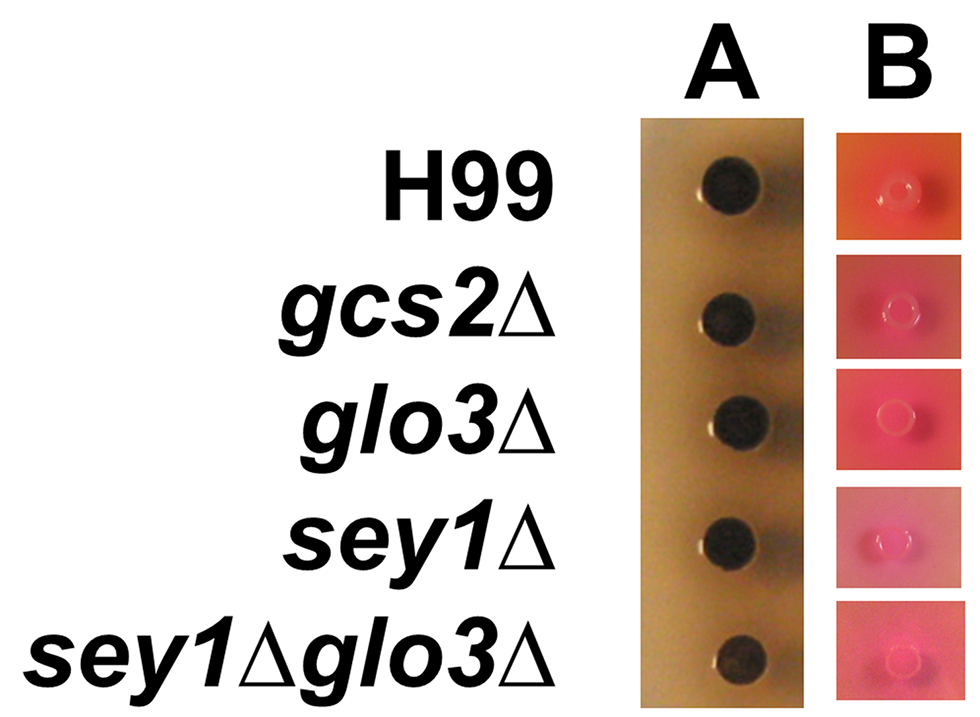

Supplement: Figure S5 — Ability to produce urease and melanin was unchanged in all mutants. Cell suspensions of each indicated strain were spotted on melanin (A) and urease (B) test media and incubated at 30°C for 2 days. (TIF) [file pone.0033022.s005.tif]

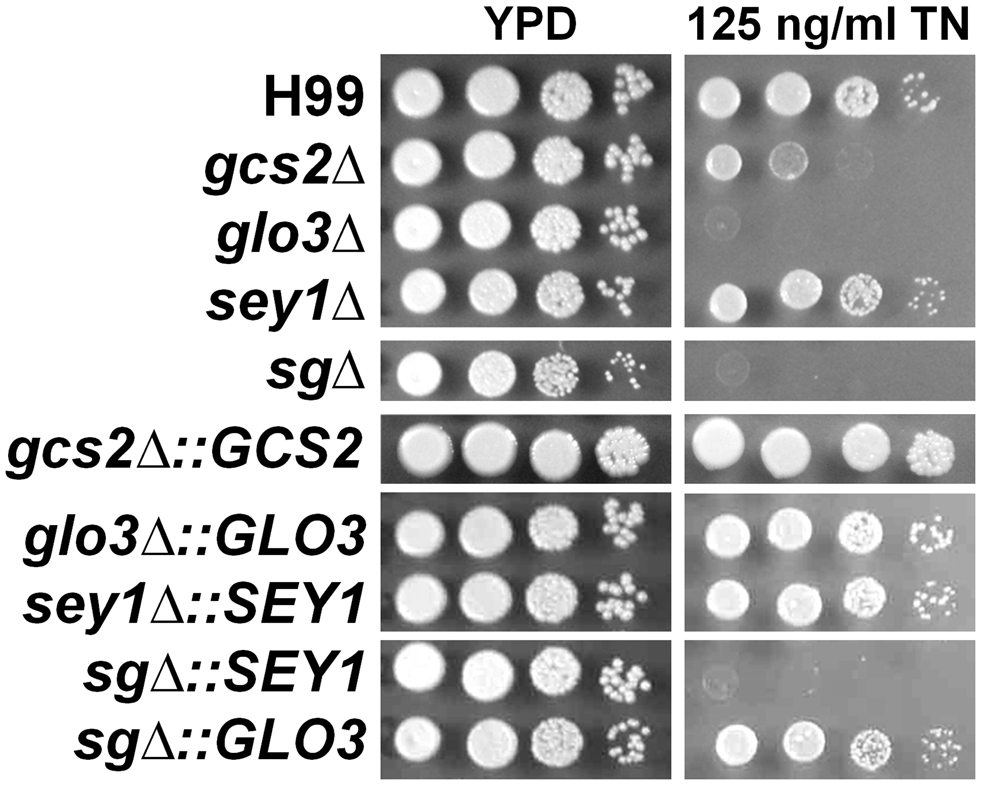

Supplement: Figure S6 — Disruption of GCS2 and GLO3 increased sensitivity to tunicamycin, an ER perturbing agent. Cells suspensions were serially diluted 10-fold from a stock at OD600 = 2 and spotted on YPD agar plates with or without 125 ng/ml tunicamycin. Plates were incubated at 30°C for 3 days. (TIF) [file pone.0033022.s006.tif]
