# Supplementary material for: Characterization of the Chromosome 4 Genes That Affect Fluconazole-Induced Disomy Formation in Cryptococcus neoformans
Source: PLoS One. 2012 Mar 7;7(3):e33022. doi: 10.1371/journal.pone.0033022 (PMC3296764; doi:10.1371/journal.pone.0033022)
Supplement: Table S1 — Primers used. (DOCX) [file pone.0033022.s007.docx]

**Table S1.** Primers used

| **Name** | **Sequence (5'-3')** | **Description** |
| --- | --- | --- |
| NatF | CATGCAGGATTCGAGTGGCATG | Amplification of *NAT*, *NEO* and *HGR* resistance cassette |
| NatR | GGAGCCATGAAGATCCTGAGGA | Amplification of *NAT*, *NEO* and *HGR* resistance cassette |
| PAI3F | ATAAGCATGCAGGATTCGAGTG | Amplification of *NAT*, *NEO* and *HGR* resistance cassette |
| PAI3R | TCGTGGTTTCAGAGACAAGGAG | Amplification of *NAT*, *NEO* and *HGR* resistance cassette |
| HABC1F1 | CACAAGCCTTTCATTCCTTTGC | Deletion of *ABC1* |
| HABC1F2 | TCCTCAGGATCTTCATGGCTCC AAACGTTCCAAGAAGGCTCA | Deletion of *ABC1* |
| HABC1R1 | CATCAGGGCGATGAAGGTTAAG | Deletion of *ABC1* |
| HABC1R2 | CATGCCACTCGAATCCTGCATG CGATCGGCTTATGTTTCCAT | Deletion of *ABC1* |
| HABC1ER | CCTTCTGCAAGGAGCAAATC | Deletion of *ABC1* |
| AMC4F1 | ATGGCCTGTGAATGTCCAGTTT | Deletion of *ABC2* |
| AMC4R1 | AACAGTTGTATCGCCGGTTTGT | Deletion of *ABC2* |
| AMC4F2 | TCCTCAGGATCTTCATGGCTCC AAATGATGGGAATGGACACG | Deletion of *ABC2* |
| AMC4R2 | CATGCCACTCGAATCCTGCATG GCTCCTAAGCGGATTTTGTG | Deletion of *ABC2* |
| AMC4EF | CCCGGTCATCCACAAATATC | Deletion of *ABC2* |
| PTC4F1 | ATCCATCTATCCGCCGACCTAT | Deletion of *PDR16* |
| PTC4R1 | CTGGCATAAATGCATGAAGACG | Deletion of *PDR16* |
| PTC4F2 | TCCTCAGGATCTTCATGGCTCC GGGAACCCTGACAGAAACAT | Deletion of *PDR16* |
| PTC4R2 | CATGCCACTCGAATCCTGCATG CGGGGTGTCAAAGAAAGAGA | Deletion of *PDR16* |
| PTC4EF | GACCTTGAAGTCGCCAGAAG | Deletion of *PDR16* |
| LROF1 | ACCGGGCTGATTGTAAGTCGTA | Deletion of *LRO1* |
| LROR1 | TCATCTCCACCAGCAGACTGAG | Deletion of *LRO1* |
| LROF2 | TCCTCAGGATCTTCATGGCTCC TGGACTGGGATCGATAGTGTC | Deletion of *LRO1* |
| LROR2 | CATGCCACTCGAATCCTGCATG GGCATAGCAAACCGTAGCAAAT | Deletion of *LRO1* |
| LROEF | GTAACTGCCGCTATTGCATTCC | Deletion of *LRO1* |
| SLC1F1 | TAACCGTCAGCATACGCACTTG | Deletion of *SLC1* |
| SLC1F2 | CTCCTTGTCTCTGAAACCACGA TTTGTTGAAGAAGCCGAAGGAG | Deletion of *SLC1* |
| SLC1R1 | CAAGAGCTCGGACAAGAGGATT | Deletion of *SLC1* |
| SLC1R2 | CACTCGAATCCTGCATGCTTAT CTGTGGCCCCTTGCAGATATAG | Deletion of *SLC1* |
| SLC1EF | ACCCCTTGGTCTCGGTTACATT | Deletion of *SLC1* |
| TOR2F1 | CTTCTCCCGACGATGAGACAGT | Deletion of *TLK1* |
| TOR2F2 | CTCCTTGTCTCTGAAACCACGA CCCACATTGGTAGTTGGCTACTG | Deletion of *TLK1* |
| TOR2R1 | CATTGCCGTTTCCCTTATACCA | Deletion of *TLK1* |
| TOR2R2 | CACTCGAATCCTGCATGCTTAT AGGCCAGGAGATCCAAGAATGT | Deletion of *TLK1* |
| TOR2EF | AGGAGTGATCGCTTCTCGTTTG | Deletion of *TLK1* |
| TOR2ER | AACCTCAAAAAGGGGGAAATGA | Deletion of *TLK1* |
| GCS1F1 | CGGCGGTATAGCAGGATACTTG | Deletion of *GCS2* |
| GCS1F2 | CTCCTTGTCTCTGAAACCACGA CATTTAAGGAGGGCATTCATGG | Deletion of *GCS2* |
| GCS1R1 | ACCCCTCAGGATGAACCAAAAT | Deletion of *GCS2* |
| GCS1R2 | CACTCGAATCCTGCATGCTTAT TGTATAGAGACGGCGATGTGGA | Deletion of *GCS2* |
| GCS1EF | GTTTGCGACGTGTATTCAGCAG | Deletion of *GCS2* |
| GCS1RRC | CACTCGAATCCTGCATGCTTATACCCCTCAGGATGAACCAAAAT | Complementation of *GCS2* |
| GLO3F1 | CCAAGATTAACGCTGGACAAGG | Deletion of *GLO3* |
| GLO3F2 | CTCCTTGTCTCTGAAACCACGA TTTCGAGGGGCGTTAATCAGTA | Deletion of *GLO3* |
| GLO3R1 | ACAGCTGCTAACGGCAAGAAAC | Deletion of *GLO3* |
| GLO3R2 | CACTCGAATCCTGCATGCTTAT GTCCATGGTGACGGAGGATAAG | Deletion of *GLO3* |
| GLO3EF | TTTGGCGTCATTTCCTTCTCAT | Deletion of *GLO3* |
| GLO3R1RC | CACTCGAATCCTGCATGCTTAT ACAGCTGCTAACGGCAAGAAAC | Complementation of *GLO3* |
| SEY1F1 | TGAGATCGTCCAACCTACGTGA | Deletion of *SEY1* |
| SEY1F2 | CTCCTTGTCTCTGAAACCACGA GAATCATCTCTGCGCATGTCTG | Deletion of *SEY1* |
| SEY1R1 | GGAGAGCTATTGCATGGTACGG | Deletion of *SEY1* |
| SEY1R2 | CACTCGAATCCTGCATGCTTAT TTATTCCGAGGTTCAGCGTGTT | Deletion of *SEY1* |
| SEY1EF | CGCAAGCCATCCAAAAGATTAG | Deletion of *SEY1* |
| SEY1R1RC | CACTCGAATCCTGCATGCTTAT GGAGAGCTATTGCATGGTACGG | Complementation of *SEY1* |
| 3CF1NOT1 | ATCGATCGATCGGCGGCCGCCTATAAGCCCCAAATCCCCAAC | Complementation of *GLO3* or *SEY1* to Chr3 |
| 3CF2AVR2 | ATCGATCGATCGCCTAGGTAGTACAAACCGCGACGCACT | Complementation of *GLO3* or *SEY1* to Chr3 |
| 3CR1XBA1 | ATCGATCGATCGTCTAGACCCATTCCCAACATCCTCTTTA | Complementation of *GLO3* or *SEY1* to Chr3 |
| 3CR2PAC1S | CGTTAATTAACAGCTCAGTGTCTTCGTGCTTT | Complementation of *GLO3* or *SEY1* to Chr3 |
| GO3EFPAC1 | ATCGATCGATCGTTAATTAATTTGGCGTCATTTCCTTCTCAT | Complementation of *GLO3* to Chr3 |
| GO3R1SAL1 | ATCGATCGATCGGTCGACACAGCTGCTAACGGCAAGAAAC | Complementation of *GLO3* to Chr3 |
| SY1EFPAC1 | ATCGATCGATCGTTAATTAACGCAAGCCATCCAAAAGATTAG | Complementation of *SEY1* to Chr3 |
| SY1R1SAL1 | ATCGATCGATCGGTCGACGGAGAGCTATTGCATGGTACGG | Complementation of *SEY1* to Chr3 |
| AIFSAL1S | CGGTCGACATAAGCATGCAGGATTCGAGTG | Complementation of *GLO3* or *SEY1* to Chr3 |
| AIRAVR2S | CGCCTAGGTCGTGGTTTCAGAGACAAGGAG | Complementation of *GLO3* or *SEY1* to Chr3 |
| 3CREF | TACTACAAGCCTCCGCCAGAAG | Complementation of *GLO3* or *SEY1* to Chr3 |
| 3CRER | CCCCTCTTCTCCTATCCGTGTT | Complementation of *GLO3* or *SEY1* to Chr3 |
| XGFP1 | ATGAGCAAGGGCGAGGAGCTGTTCAC | Transformation of Sec61β-GFP |
| XGFP2 | CTTGTACAGCTCGTCCATGCCGT | Transformation of Sec61β-GFP |
| SECBF1 | GCGGATCC CTCCACTGAGCCACTTCTCC | Transformation of Sec61β-GFP |
| SECBF2X | ACGGCATGGACGAGCTGTACAAGTAGGCATTCCTGGAATAGCTTGG | Transformation of Sec61β-GFP |
| SECBF3 | GCGCGGCCGC GCATTCCTGGAATAGCTTGG | Transformation of Sec61β-GFP |
| SECBF4 | GTGACCTTGGCAAATGAGGT | Transformation of Sec61β-GFP |
| SECBR1 | GCGGGCCC TGGAACGAGAGATTGTGCTG | Transformation of Sec61β-GFP |
| SECBR2 | GCAAGCTT TGGAACGAGAGATTGTGCTG | Transformation of Sec61β-GFP |
| SECBR3 | GTGAACAGCTCCTCGCCCTTGCTCAT GTTGGTAAAAGCCCTGATGA | Transformation of Sec61β-GFP |
| SECBF2AVR2 | ATCGCCTAGGCATTCCTGGAATAGCTTGG | Transformation of Sec61β-GFP |
| XGFP2AVR2 | ATCGCCTAGGTGTACAGCTCGTCCATGCCGT | Transformation of Sec61β-GFP |
| SECBEF | GGCAGAACGAACCACTTGTACC | Transformation of Sec61β-GFP |
| SECBER | CTTATTGGGCTCCAGCATATCG | Transformation of Sec61β-GFP |
